# Supplementary material for: The deubiquitylase OTUD3 stabilizes GRP78 and promotes lung tumorigenesis
Source: Nat Commun. 2019 Jul 2;10:2914. doi: 10.1038/s41467-019-10824-7 (PMC6606649; doi:10.1038/s41467-019-10824-7)
Supplement: Supplementary file 1 — Supplementary Information [file 41467_2019_10824_MOESM1_ESM.pdf]

The deubiquitylase OTUD3 stabilizes GRP78 and promotes lung tumorigenesis

Du et al.

Supplementary Figures

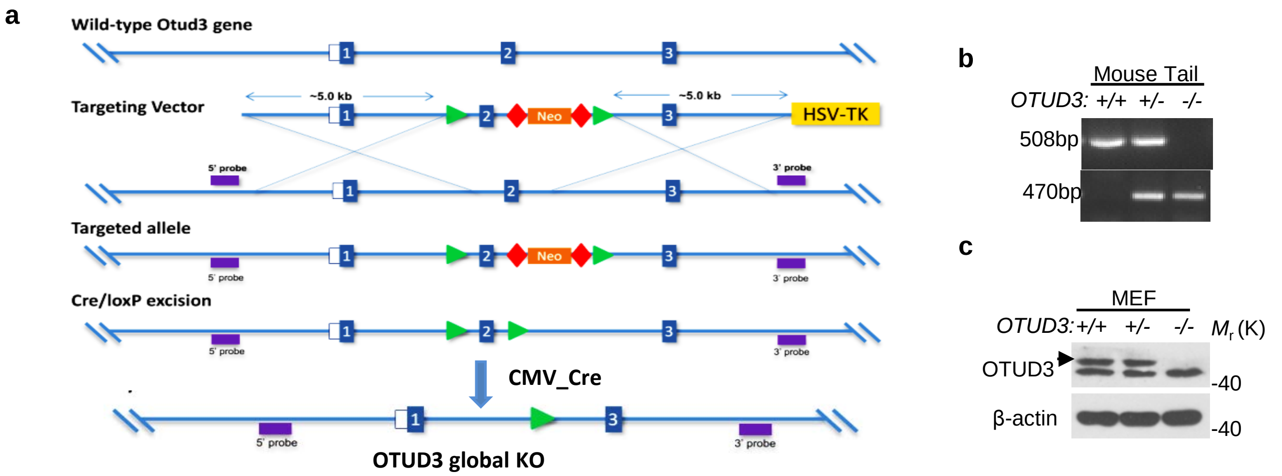

d

Genotype	E14.5	E16.5	E18.5	P0	P7
OTUD3$^{+/+}$	7	6	12	75	73
OTUD3$^{+/-}$	14	13	31	151	170
OTUD3$^{-/-}$	6	7	14	72	72
Total	27	26	57	298	295

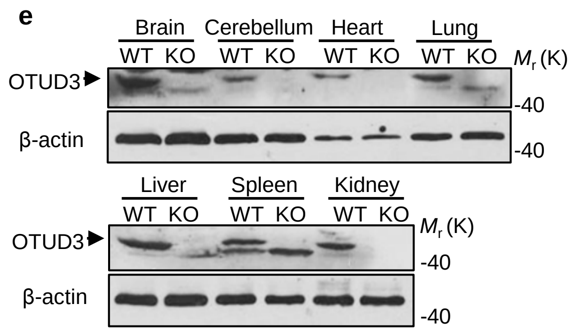

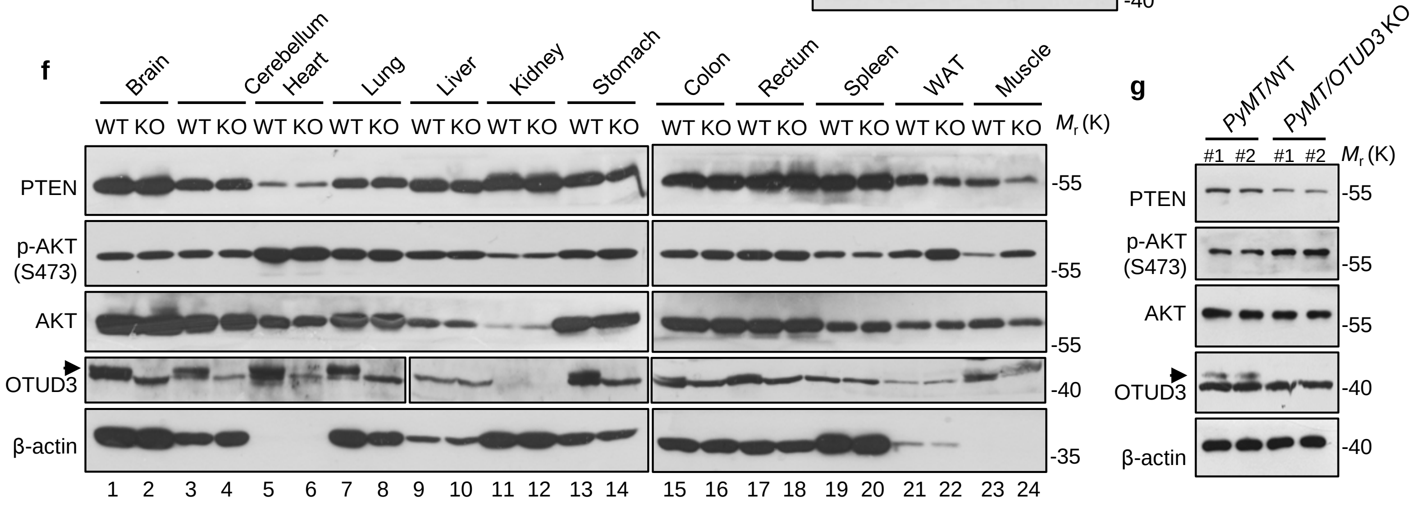

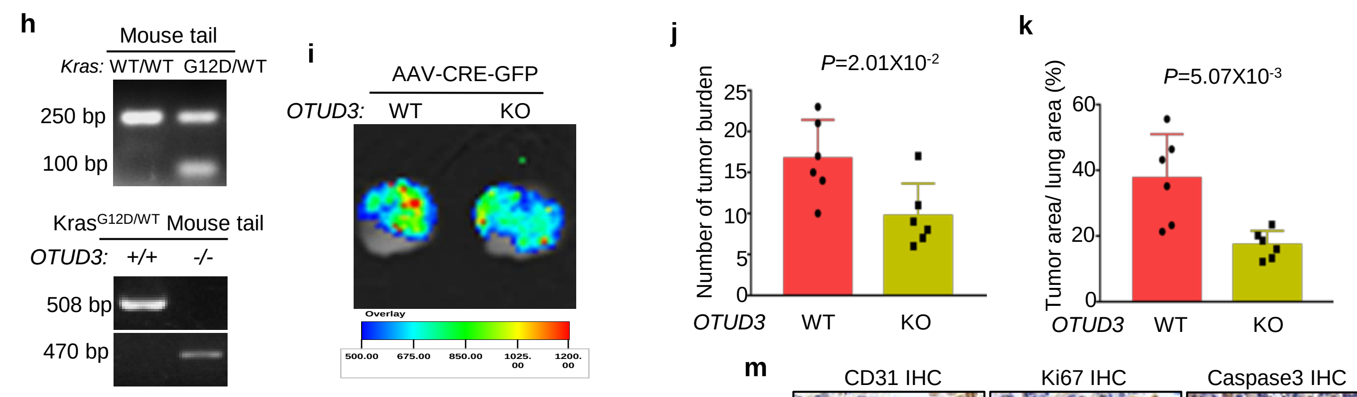

l

	Tumor grade-mean number of tumor ±SEM			
	G Ⅰ	G Ⅱ	GⅢ	GⅣ
OTUD3 WT	1.667 ±0.667	3.5 ±0.5	4.667±0.4216	5.167±1.138
OTUD3 KO	3.833 ±1.108	3.5 ±0.4282	1.167±0.4773	1.167±0.6009
P value	1.25X10^{-1}	9.99X10^{-1}	3.00X10^{-4}	1.11X10^{-2}

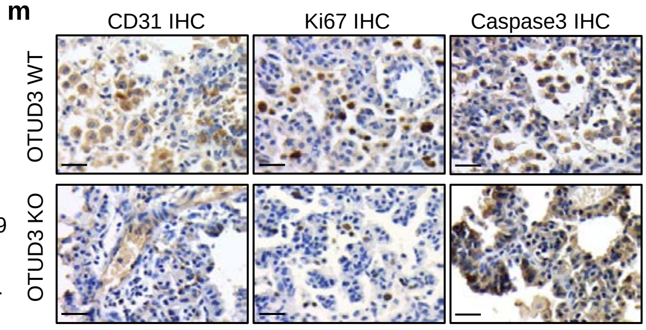

Supplementary Figure 1 Generation of *OTUD3* knockout, *Kras*$^{LSL\text{-}G12D/WT}$/*OTUD3* WT and *Kras*$^{LSL\text{-}G12D/WT}$/*OTUD3* KO mice**. a**, The strategy for generation of *OTUD3* knockout mice. **b**, Genotyping of *OTUD3* knockout mice by PCR. **c**, The expression levels of OTUD3 in *OTUD3* WT and KO MEFs were detected by western blotting. **d,** The number of *OTUD3*$^{+/+}$, *OTUD3*$^{+/-}$ and *OTUD3*$^{-/-}$ mice at the indicated stages was calculated. **e,** Immunoblotting of OTUD3 in different tissues from adult *OTUD3* WT and KO littlemates (2 months) verified the OTUD3 protein deficient effect in mice, WAT, white adipose tissue. **f,** Immunoblotting of p-AKT(S473), AKT and PTEN in tissues from *OTUD3* WT and KO littlemates (2 months). **g**, PTEN and p-AKT (S473) levels in breast cancer tissues from *PyMT/*WT and *PyMT/OTUD3* KO mice. **h,** PCR was used to Genotype the *Kras*$^{LSL\text{-}G12D/t}$/*OTUD3* WT and *Kras*$^{LSL\text{-}G12D/t}$/*OTUD3* mice. **i,** The efficiency of AAV-mediated gene delivery was evaluated by bioluminescence imaging. **j,** Show a quantification of tumor number in indicated planes from *OTUD3* WT or KO mice (*n*=6). The data are shown as mean $\pm$ s.d. Student's *t*-test. **k,** Show a quantification of tumor area in indicated planes from *OTUD3* WT or KO mice (*n*=6). The data are shown as mean $\pm$ s.d. Student's *t*-test. **l,** Table of tumor grade statistics from *OTUD3* WT or KO mice (*n*=6). **m,** Immunohistochemistry analysis of protein levels of Ki-67, CD31 and cleaved Caspase-3 in lung tumors of *Kras*$^{G12D/WT}$/*OTUD3* WT and *Kras*$^{G12D/WT}$/*OTUD3* KO mice, respectively (Scale bar, 50 μm). For **c**,**e**,**f**,**g,** results are representative of three independent experiments. Uncropped images of blots are shown in Supplementary Fig. 9.

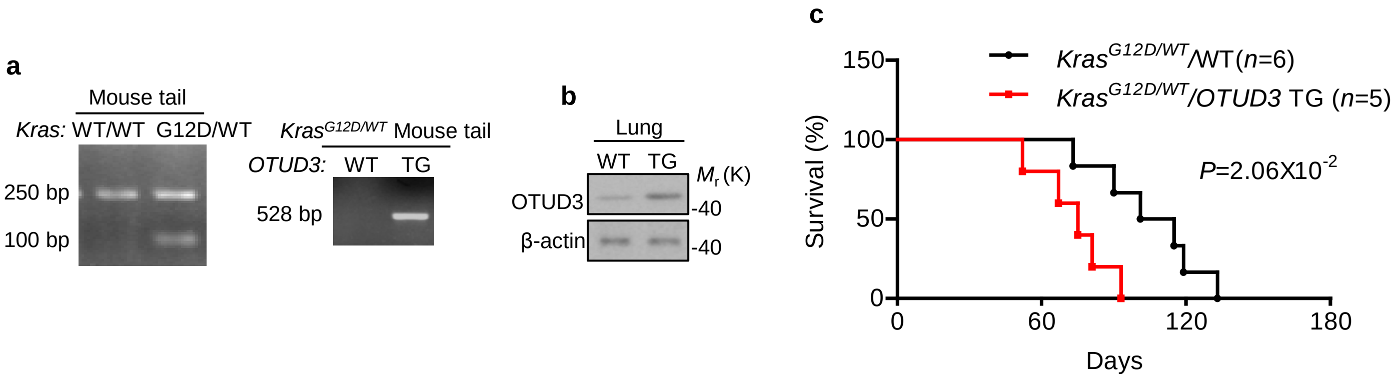

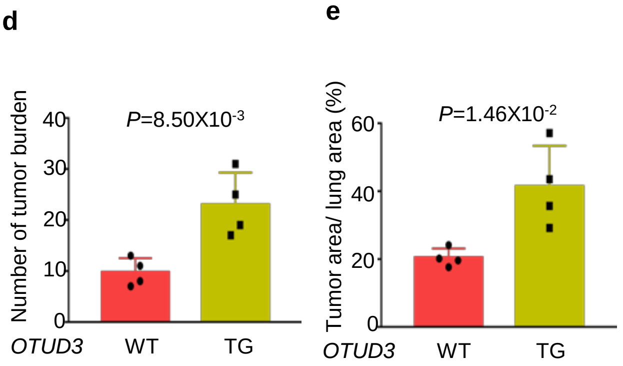

f

	Tumor grade-mean number of tumor ±SEM			
	G I	G II	GIII	GIV
OTUD3 WT	2.75 ±0.8539	3.5 ±0.5	2.75±0.4787	0.75±0.25
OTUD3 TG	1.75 ±0.4787	4.5 ±0.2887	7.5±1.19	9±2.273
P value	3.46X10-1	1.34 X10-1	1.01 X10-2	1.13 X10-2

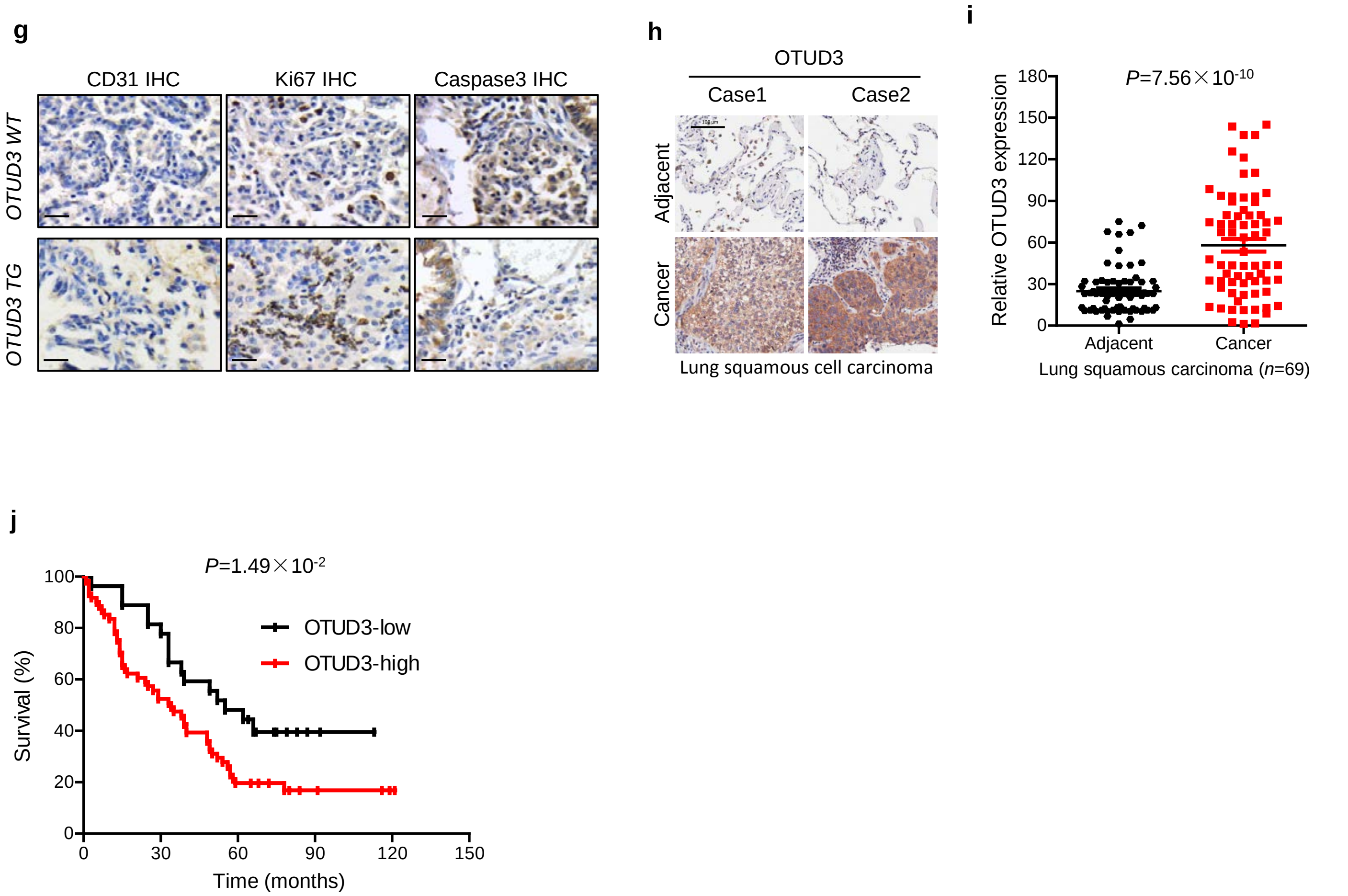

Supplementary Figure 2 Quantification of tumor number, tumor area, tumor grade and overall survival of lung cancer patients with OTUD3 expression. **a,** PCR was used to confirm the generation of *Kras*$^{G12D/WT}$/*OTUD3* TG mice. **b,** The OTUD3 expression levels in *Kras*$^{G12D/WT}$/*OTUD3* WT and *Kras*$^{G12D/WT}$/*OTUD3* TG mice were detected. **c,** Kaplan–Meier plot showing overall survival of mice with the indicated genotypes infected with AdCre (*Kras*$^{G12D/WT}$/*OTUD3* WT, n=6; *Kras*$^{G12D/WT}$/*OTUD3* TG, n=5), log-rank test. **d,** Show a quantification of tumor number in indicated planes from *OTUD3* WT or TG mice (n=4). The data are shown as mean $\pm$ s.d. Student's t-test. **e,** Show a quantification of tumor area in indicated planes from *OTUD3* WT or TG mice (n=4). The data are shown as mean $\pm$ s.d. Student's t-test. **f,** Table of tumor grade statistics from *OTUD3* WT and TG mice (n=4). **g,** Immunohistochemistry analysis of protein levels of Ki-67, CD31 and cleaved Caspase-3 in lung tumors of *Kras*$^{G12D/WT}$/*OTUD3* WT and *Kras*$^{G12D/WT}$/*OTUD3* TG mice, respectively (Scale bar, 50 μm). **h,** Representative images from immunohistochemical staining of OTUD3 in human lung squamous carcinoma (Scale bar, 100 μm). **i,** Whisker plots show the OTUD3 expression in primary lung squamous carcinoma (n=69). Data were analysed using Mann–Whitney test. **j,** Kaplan–Meier plot of overall survival of 88 patients with lung adenocarcinoma. Statistical significance was calculated using log-rank test. Statistics source data can be found in Supplementary Data 1.

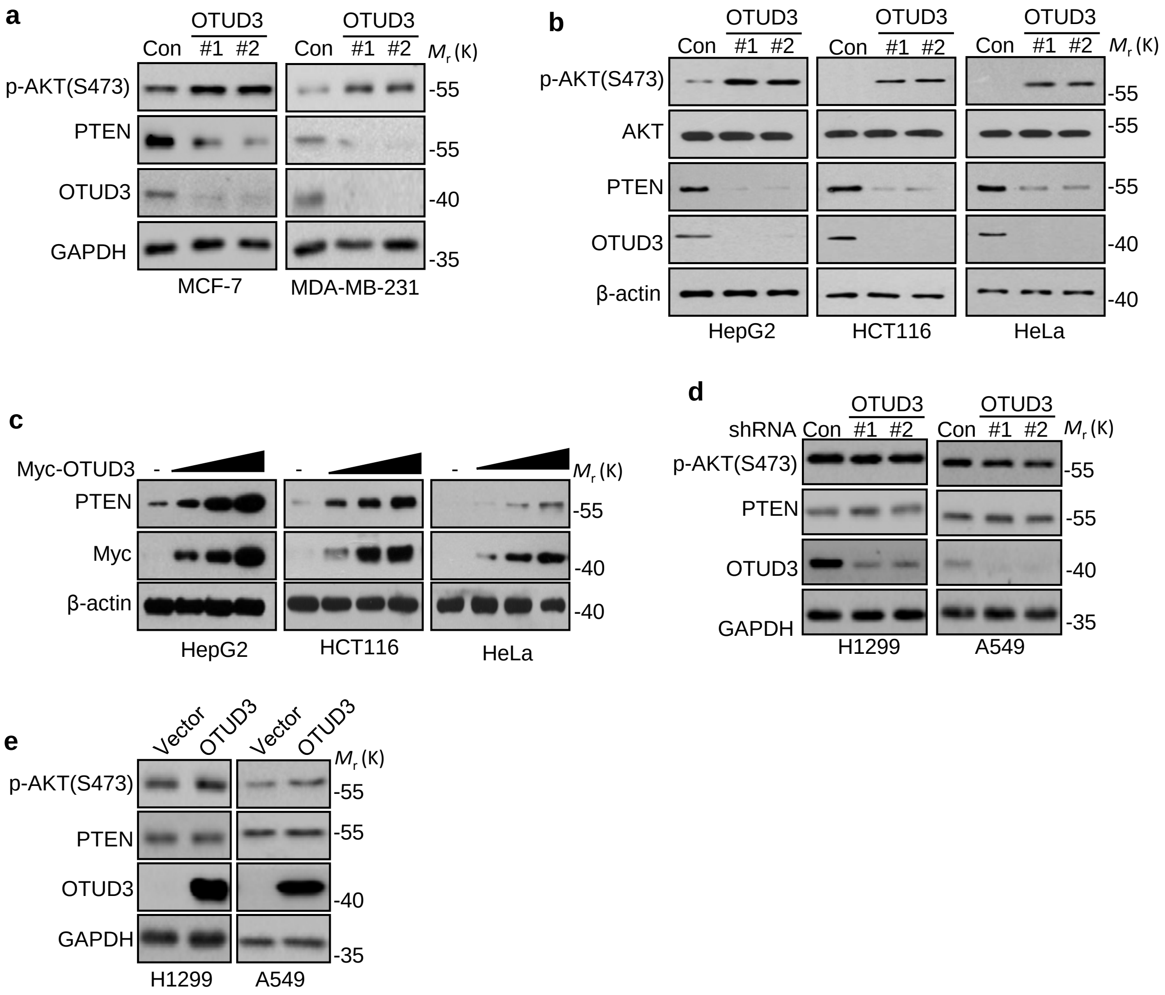

Supplementary Figure 3 Comparison of the effect of OTUD3 on PTEN expression in different cancer cells. **a-b**, OTUD3 was knockdown in the indicated cancer cell lines. The protein levels of OTUD3, PTEN and p-AKT(S473) were measured by western blotting. **c,** Increasing amounts of OTUD3 WT was transfected into the indicated cancer cell lines and PTEN expression was detected. **d-e,** The protein levels of p-AKT(S473), PTEN and OTUD3 were measured in OTUD3-deleted or OTUD3-overexpressing lung cancer cells H1299 and A549. For all panels, results are representative of three independent experiments. Uncropped images of blots are shown in Supplementary Fig. 9.

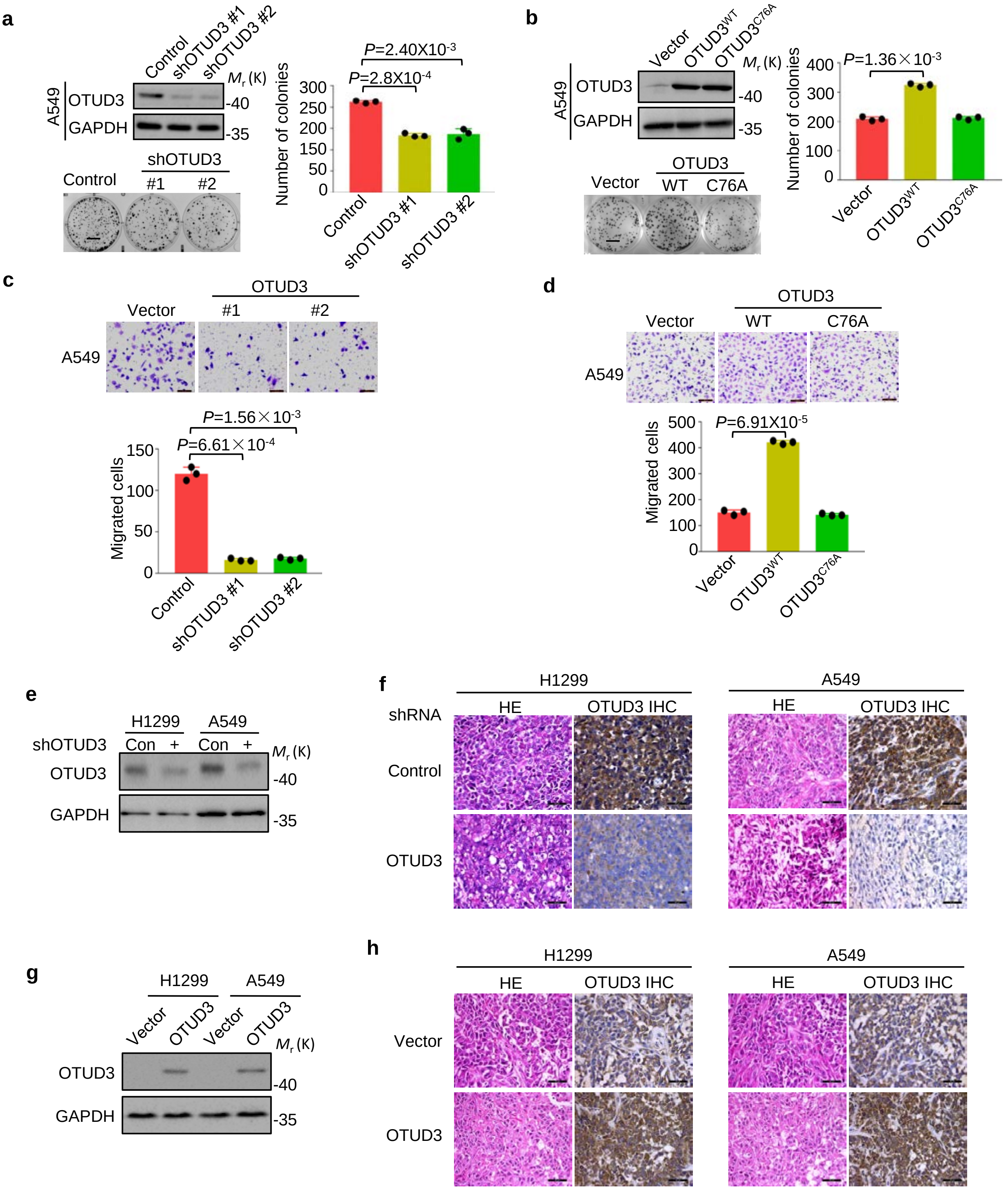

Supplementary Figure 4 OTUD3 promotes lung cancer cell proliferation and migration. **a**, OTUD3 was knocked down in lung cancer cell lines A549 using shRNA. The protein levels of OTUD3 were analyzed by western blotting. Effects of OTUD3 on the cell growth were examined by colony formation assay. Results are representative of *n*=3 independent experiments (Scale bar, 1 cm). **b**, OTUD3 WT or OTUD3 C76A were overexpressed in A549. The protein levels of OTUD3 WT and OTUD3 C76A were analyzed by western blotting. Effects of OTUD3 WT or C76A on the cell growth were examined by colony formation assay. Results are representative of *n*=3 independent experiments (Scale bar, 1 cm). **c**, OTUD3 was knocked down in lung cancer cell lines A549 through shRNA. Effect of OTUD3 on the A549 cells migration was examined by transwell assay. Results are representative of *n*=3 independent experiments (Scale bar, 100 µm). **d**, Effects of OTUD3 WT or C76A on the A549 cells migration was examined by transwell assay. Results are representative of *n*=3 independent experiments (Scale bar, 100 µm). **e**, OTUD3 shRNA-transduced H1299 cells and A549 cells were established.

f, The protein levels of OTUD3 were analysed by IHC in tumor formation in figure 4f and figure 4h (Scale bar, 50 μm). **g**, H1299 cells and A549 cells with or without stablely overexpressing OTUD3 were established. **h**, The protein levels of OTUD3 were analyzed by IHC in tumor formation figure 4**g** and figure 4**i** (Scale bar, 50 μm). For panels **a**-**d**, data are shown as mean $\pm$ s.d. Student's *t*-test. Statistics source data can be found in Supplementary Data 1. Uncropped images of blots are shown in Supplementary Fig. 9.

a

No.	Data base ID	Protein Name
1	NM_002473.5	Homo sapiens myosin, heavy chain 9, non-muscle (MYH9)
2	NM_004559.3	Homo sapiens Y box binding protein 1 (YBX1)
3	NM_001357.4	Homo sapiens DEAH (Asp-Glu-Ala-His) box helicase 9 (DHX9)
4	NM_005347.4	Homo sapiens heat shock 70kDa protein 5 (glucose-regulated protein, 78kDa) (GRP78)
5	NM_003604.2	Homo sapiens insulin receptor substrate (IRS4)
6	NM_005082.4	Homo sapiens tripartite motif containing 25 (TRIM25)
7	NM_001039465.1	Homo sapiens serine/arginine-rich splicing factor 5 (SRSF5)
8	NM_001102653.1	Homo sapiens OTU deubiquitinase 4 (OTUD4)
9	NM_004739.3	Homo sapiens metastasis associated 1 family, member 2 (MTA2)
10	NM_005381.2	Homo sapiens nucleolin (NCL)

The list of OTUD3 binding protein identified by IP-MS

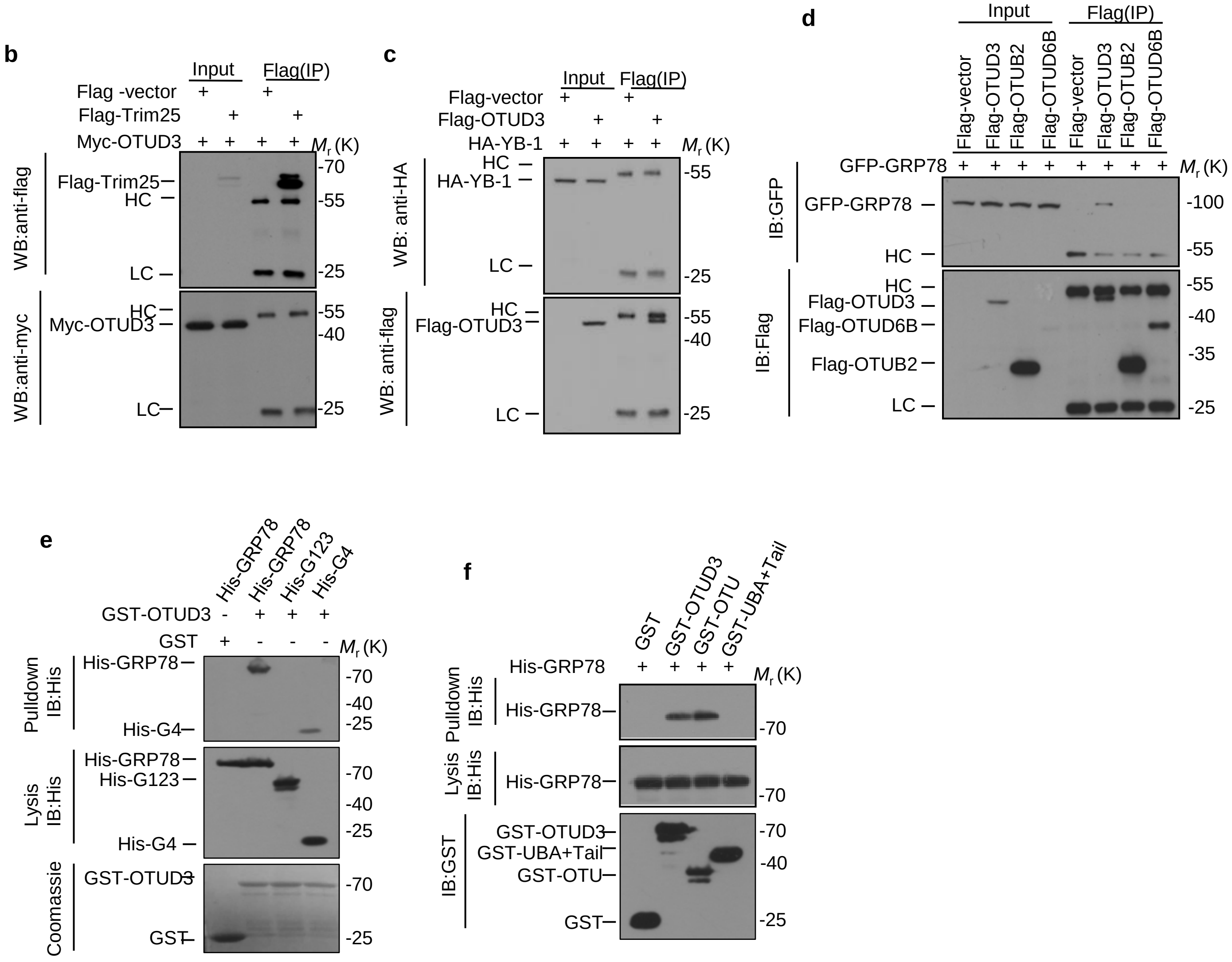

Supplementary Figure 5 Identification of the OTUD3 interacting proteins. **a,** The proteins potentially interact with OTUD3 were listed in the table. **b**, Myc-OTUD3 and Flag-Trim25 were co-transfected into 293T cells. Cell lysates were immunoprecipitated with anti-Flag antibody. Immunoprecipitates were analysed by immunoblotting with anti-Myc and anti-Flag antibodies. **c,** Flag-OTUD3 and HA-YB-1 were co-transfected into 293T cells. Cell lysates were immunoprecipitated with anti-Flag antibody. Immunoprecipitates were analysed by immunoblotting with anti-HA and anti-Flag antibodies. **d,** 293T cells were transfected with the indicated Flag–DUBs were subjected to immunoprecipitation (IP) with anti-Flag antibodies. **e**, GST pull-down assays were performed to indicate the direct interaction between OTUD3 and GRP78 truncations. **f**, GST pull-down assays were performed to indicate the direct interaction between GRP78 and OTUD3 truncations. All panels are representative results of three independent experiments. The lysates and immunoprecipitates were analysed. Uncropped images of blots are shown in Supplementary Fig. 9.

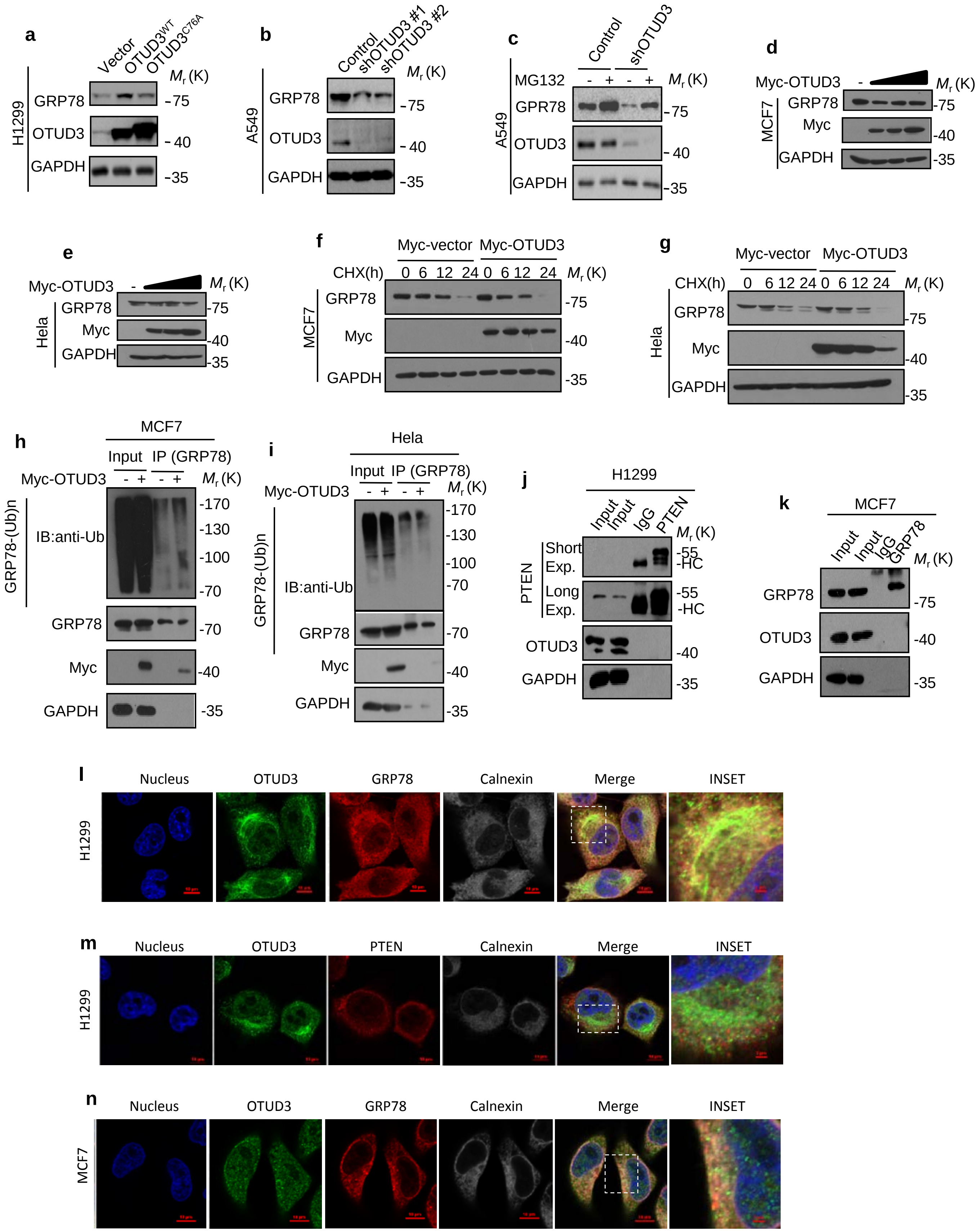

Supplementary Figure 6 Comparison of the effect of OTUD3 on GRP78 expression in different cancer cells. **a,** OTUD3 WT and OTUD3 C76A were overexpressed into H1299 cells. Cell lysates were subject to detect with the indicated antibodies. **b**, OTUD3 was depleted in the A549 cells. Cell lysates were subjected to detect with the indicated antibodies. **c,** A549 cells with or without OTUD3 knockdown were treated with MG132 for 8 h. Cell lysates were subject to analyze with the indicated antibodies. **d,e,** Increasing amounts of OTUD3 was transfected

into MCF7 cells (**d**) and HeLa cells (**e**). The GRP78 protein levels were detected. **f,g,** MCF7 and HeLa cells with or without overexpressing OTUD3 were treated with CHX (10 µg/ml). The half-life of GRP78 were measured. **h,i,** The indicated constructs were transfected into MCF7 and HeLa cells. The cells were then treated with MG132 for 8 h before collection. The whole-cell lysate Cell lysates were immunoprecipitated with anti-GRP78 antibody. The ubiquitylation of GRP78 was detected. **j,k** H1299 and MCF7 cell lysates were immunoprecipitated with anti-PTEN and anti-GRP78 antibodies, immunoprecipitates were analysed using the indicated antibodies. **l,m**, Confocal analysis of OTUD3 (488, green) co-localization with GRP78 or PTEN (594, red) and Calnexin（647，grey）in H1299 cells. Scale bars, 10 µm. The insets show higher magnification (Scale bars, 2 µm). Nuclei were stained with DAPI. **n**, Confocal analysis of OTUD3 (488, green) co-localization with GRP78 (594, red) and Calnexin（647, grey）in MCF7 cells. Scale bars, 10 µm. The insets show higher magnification (Scale bars, 2 µm). Nuclei were stained with DAPI. For all panels, data are representative results of three independent experiments. Uncropped images of blots are shown in Supplementary Fig. 9.

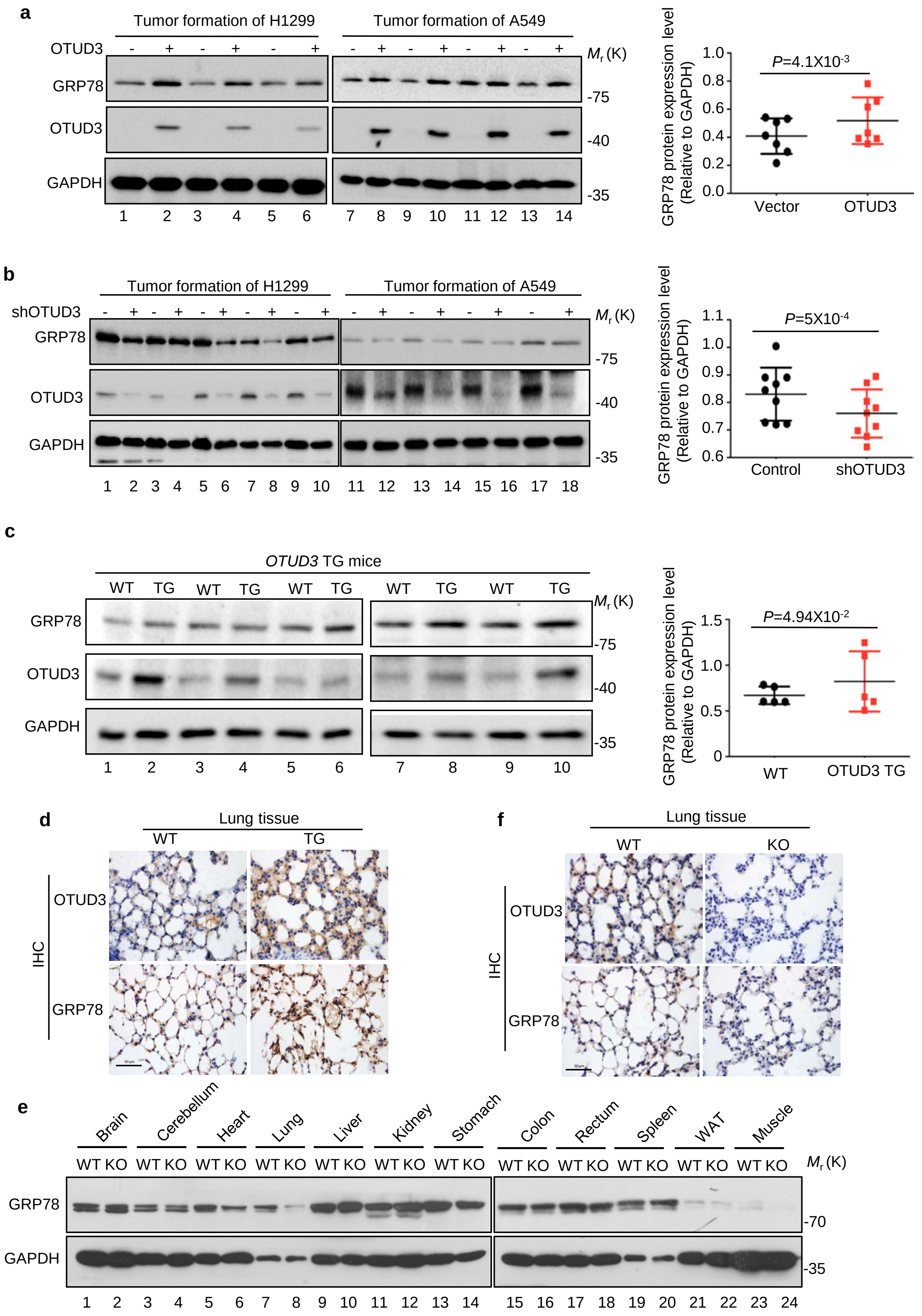

a
Tumor formation of H1299
Tumor formation of A549
OTUD3
GRP78
OTUD3
GAPDH
M_r (K)
-75
-40
-35
1 2 3 4 5 6 7 8 9 10 11 12 13 14
GRP78 protein expression level (Relative to GAPDH)
P=4.1X10-3
Vector
OTUD3
b
Tumor formation of H1299
Tumor formation of A549
shOTUD3
GRP78
OTUD3
GAPDH
M_r (K)
-75
-40
-35
1 2 3 4 5 6 7 8 9 10 11 12 13 14 15 16 17 18
GRP78 protein expression level (Relative to GAPDH)
P=5X10-4
Control
shOTUD3
c
OTUD3 TG mice
WT TG WT TG WT TG WT TG WT TG
GRP78
OTUD3
GAPDH
M_r (K)
-75
-40
-35
1 2 3 4 5 6 7 8 9 10
GRP78 protein expression level (Relative to GAPDH)
P=4.94X10-2
WT
OTUD3 TG
d
Lung tissue
WT
TG
IHC
OTUD3
GRP78
f
Lung tissue
WT
KO
IHC
OTUD3
GRP78
e
Brain
Cerebellum
Heart
Lung
Liver
Kidney
Stomach
Colon
Rectum
Spleen
WAT
Muscle
WT KO WT KO WT KO WT KO WT KO WT KO WT KO WT KO WT KO WT KO WT KO WT KO
M_r (K)
GRP78
GAPDH
-70
-35
1 2 3 4 5 6 7 8 9 10 11 12 13 14 15 16 17 18 19 20 21 22 23 24

Supplementary Figure 7 Analysis of the association between OTUD3 and GRP78 in tumor and lung tissue. **a**, The protein levels of GRP78 and OTUD3 were analysed by western blotting in tumor formation from OTUD3 overexpressing cells (figure 4g and figure 4i). For the band intensity of GRP78 protein expression level in tumors, Vector, *n*=7; OTUD3, *n*=7. **b**, The protein levels of GRP78 and OTUD3 were analyzed by western blotting in tumor formation from OTUD3 depleted cells (figure 4f and figure 4h). For the band intensity of GRP78 protein expression level in tumors, Vector, *n*=9; OTUD3, *n*=9. **c**, The protein levels of GRP78 were analysed by western blotting in lung tissue of *OTUD3* WT and TG mice (*n*=5). **d**, Immunohistochemistry analysis protein levels of OTUD3 and GRP78 in the lung tissue of *OTUD3* WT and TG mice (Scale bar, 50 μm). **e**, Immunoblotting of GRP78 in tissues from *OTUD3* WT and KO littlemates (2 months). **f**, Immunohistochemistry analysis protein levels of OTUD3 and GRP78 in the lung tissue of *OTUD3* WT and KO mice (Scale bar, 50 μm). Panel **e** is representative result of three independent experiments. In panel **a**-**c**, data are shown as mean ± s.d; Student's *t*-test. Statistics source data can be found in Supplementary Data 1. Uncropped images of blots are shown in Supplementary Fig. 9.

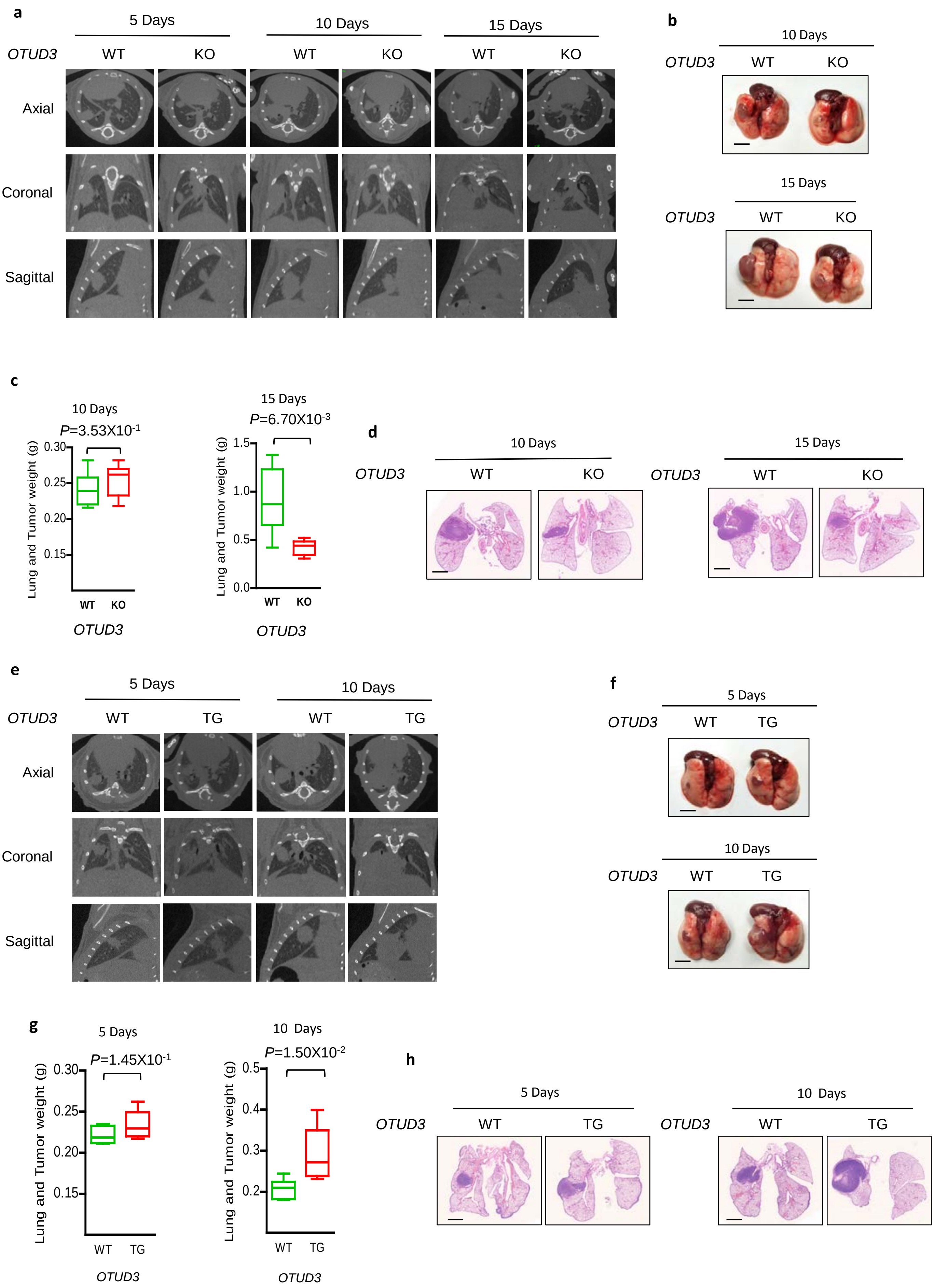

a
5 Days
10 Days
15 Days
OTUD3
WT
KO
Axial
Coronal
Sagittal
b
10 Days
15 Days
c
P=3.53X10-1
P=6.70X10-3
Lung and Tumor weight (g)
d
e
TG
f
g
P=1.45X10-1
P=1.50X10-2
h

Supplementary Figure 8 Effect of OTUD3 deficiency or overexpression on tumor microenvironment during tumorigenesis. **a**, Micro-CT images in indicated planes in the 5, 10 and 15 days after orthotopically transplant Lewis lung carcinoma cells in *OTUD3* WT or KO mice . **b,** Representative images of lung lesions from the indicated experimental groups in the 10 and 15 days (Scale bar, 0.5 cm). **c,** The weight of lung and tumor (*n*=6) in the 10 and 15 days. Data are shown as mean ± s.d; Student's *t*-test. For the box and whisker graphs in c, the box extends from the 25th to the 75th percentile, the line inside the box is the median and the whiskers are the fifth and 95th percentiles. **d,** Representative images of lung H&E staining from the indicated experimental groups (Scale bar, 0.5 cm). **e**, Micro-CT images in indicated planes in the 5 and 10 days after orthotopically transplant Lewis lung carcinoma cells in *OTUD3* WT or TG mice. **f,** Representative images of lung lesions from the indicated experimental groups in the 5 and 10 days (Scale bar, 0.5 cm). **g,** The weight of lung and tumor (*n*=6) in the 5 and 10 days. Data are shown as mean ± s.d; Student's *t*-test. For the box and whisker graphs in g, the box extends from the 25th to the 75th percentile, the line inside the box is the median and the whiskers are the fifth and 95th percentiles. **h,** Representative images of lung H&E staining from the indicated experimental groups (Scale bar, 0.5 cm).

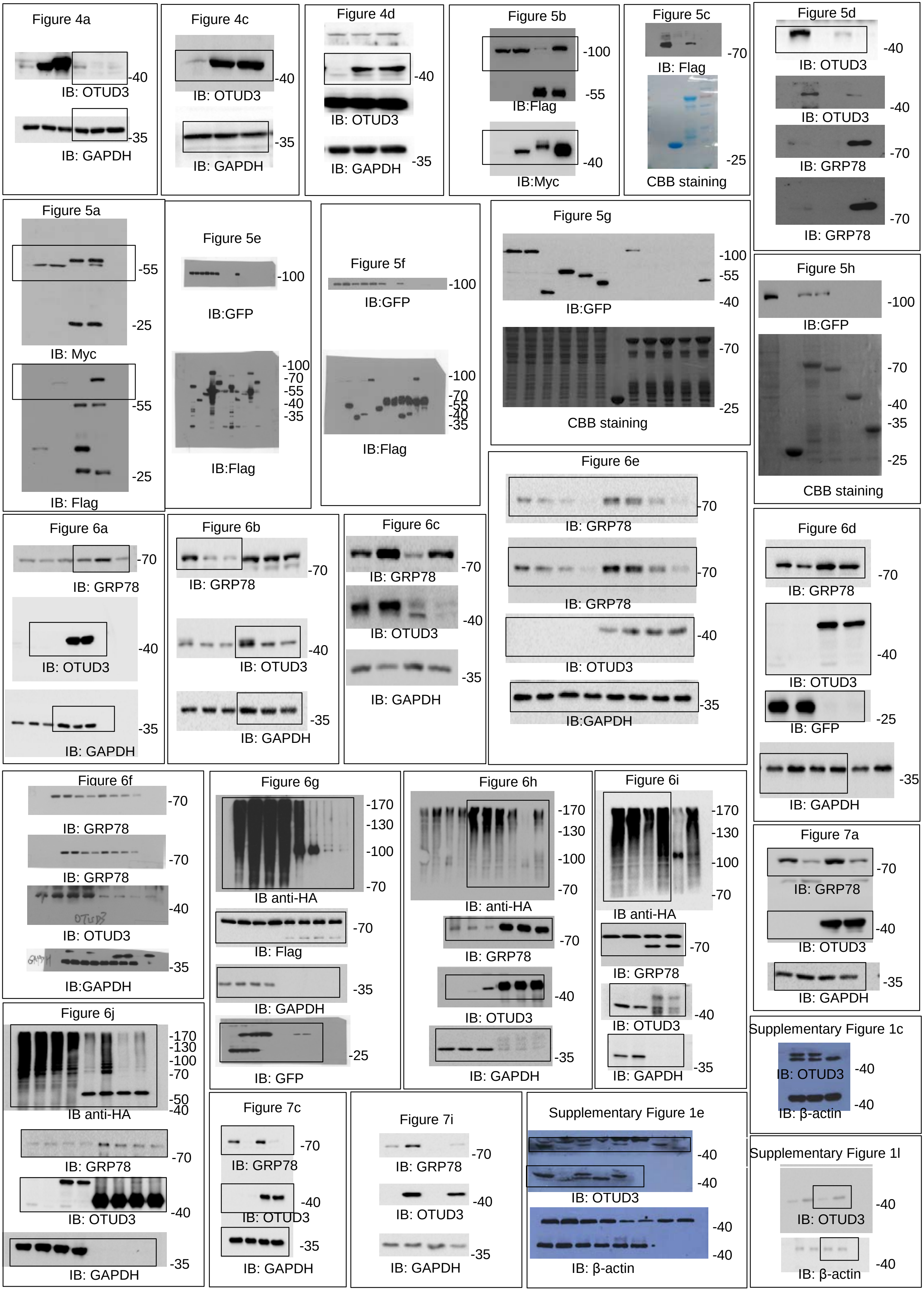

Supplementary Figure 9 Uncropped scans of Western blots.

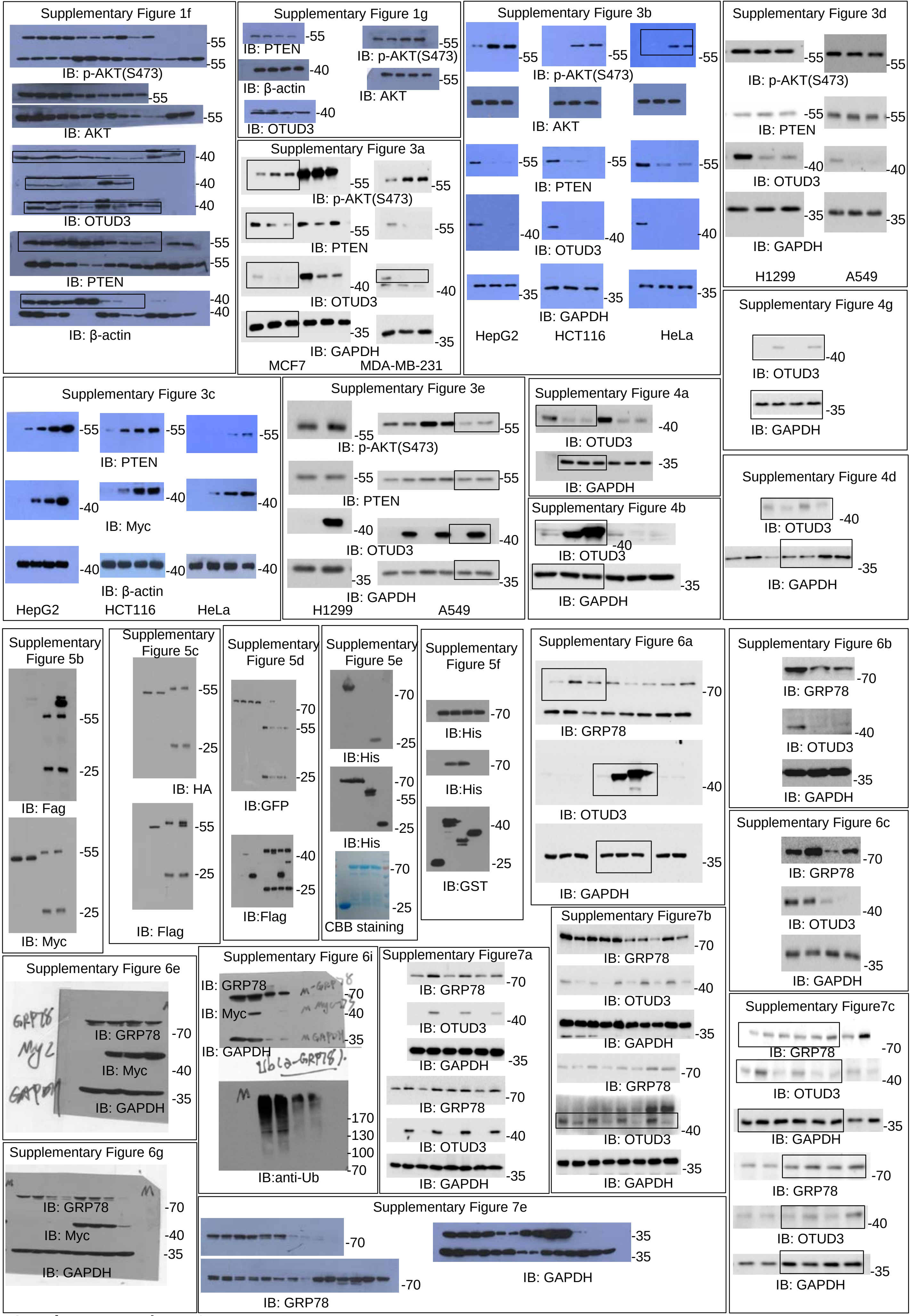

Supplementary Figure 9 Continued

Supplementary Tables

Supplementary Table 1: The primers used for the indicated gene products

Genes	Forward	Reverse	product
OTUD3 KO mice	CACGATGCTTGACAGTGGCGTTG	CCGACTGAGTTCGTCTAACACG	508 bp
	CACGATGCTTGACAGTGGCGTTG	AGTCCCATCATCCTCAGACAGC	470 bp
OTUD3 TG mice	GGCCGCTAATACGACTCACT	TTCTGTGGCTGAAAACCCCT	528 bp
MMTV-PyMT mice	GGAAGCAAGTACTTCACAAGGG	GGAAAGTCACTAGGAGCAGGG	556 bp
*KrasLSL-G12D/*WT mice	TGTCTTTCCCCAGCACAGT	GCAGGTCGAGGGACCTAATA	250 bp
	CTGCATAGTACGCTATACCCTGT	CTGCATAGTACGCTATACCCTGT	100 bp

Supplementary Table 2: Target sequences of shRNAs for individual genes

	**shRNA targeting OTUD3**
no.1	5-TGGAAATCAGGGCTTAAAT-3
no.2	5-GAGTTACACATCGCATATC-3
	**shRNA targeting GRP78**
no.1	5-CTTGTTGGTGGCTCGACTCGA-3
no.2	5-AGATTCAGCAACTGGTTAAAG-3
