# Supplementary material for: The deubiquitylase OTUD3 stabilizes GRP78 and promotes lung tumorigenesis
Source: Nat Commun. 2019 Jul 2;10:2914. doi: 10.1038/s41467-019-10824-7 (PMC6606649; doi:10.1038/s41467-019-10824-7)
Supplement: Supplementary file 3 — Description of Additional Supplementary Files [file 41467_2019_10824_MOESM3_ESM.docx]

Description of Additional Supplementary Files

**Supplementary Data 1:** Statistics source data.

**Supplementary Data 2**: IP-MS data of potential OTUD3-interacting proteins.
